# Analysis of the Biomechanical Behavior of an Animal Model of Triple Hamstring Graft Configuration for Combined ACL and ALL Reconstruction with a Single Femoral Tunnel and a Single Strand for ALL Reconstruction

**DOI:** 10.1055/s-0044-1785664

**Published:** 2024-05-19

**Authors:** Maria Luzete Costa Cavalcante, Renata Clazzer, Camilo Partezani Helito, Rodrigo Nogueira de Codes, Lana Lacerda de Lima, Diego Ariel de Lima

**Affiliations:** 1 Departamento de Cirurgia, Programa de Pós-Graduação em Ciências Médico-Cirúrgicas, Universidade Federal do Ceará (UFC), Fortaleza, CE, Brasil; 2 Departamento de Ortopedia e Traumatologia, Instituto de Ortopedia e Traumatologia, Hospital das Clínicas, Faculdade de Medicina, Universidade de São Paulo, São Paulo, SP, Brasil; 3 Departamento de Engenharia e Tecnologia, Centro de Engenharias, Universidade Federal Rural do Semi-Árido, Mossoró, RN, Brasil; 4 Departamento de Ciências da Saúde, Centro de Ciências Biológicas e da Saúde, Universidade Federal Rural do Semi-Árido, Mossoró, RN, Brasil

**Keywords:** anterior cruciate ligament, anterior cruciate ligament reconstruction, knee joint, ligaments, articular

## Abstract

**Objective** To describe and biomechanically test a configuration, in an animal model that simulates the triple hamstring graft for combined anterior cruciate ligament (ACL) and anterolateral ligament (ALL) reconstruction, with a single femoral tunnel and a single strand for ALL reconstruction.

**Methods** Deep flexor porcine tendons were used as the graft and fixed with titanium interference screws in a polyurethane block. The samples were divided into 3 groups: group 1 (control) – with a quadruple graft; group 2–with a simple triple graft; and group 3–with a braided triple graft. The tests were conducted using an EMIC DL 10000 (Instron Brasil Equipamentos Científicos Ltda., São José dos Pinhais, PR, Brazil) electromechanical universal testing machine.

**Results** The samples in groups 1, 2, and 3 obtained mean peak forces of 816.28 ± 78.78 N, 506.95 ± 151.30 N, and 723.16 ± 316.15 N, respectively. In Group 3, braiding increased graft diameter by 9% to 14%, and caused a shortening of 4% to 8% compared with group 1, with an average peak force increase of ∼ 200 N ( *p* < 0.05). Regarding peak forces, there was no statistically significant difference between groups 1 and 3, indicating that quadruple and braided triple grafts showed similar strength results.

**Conclusion** The triple-braided hamstring graft configuration for combined ACL and ALL reconstruction with a single femoral tunnel and a single strand for ALL reconstruction may become a biomechanically viable solution, with potential clinical application.

## Introduction

Combined reconstruction of the anterior cruciate ligament (ACL) and the anterolateral ligament (ALL) of the knee has shown excellent results in specific patient groups. 1 2 3 4 It may reduce graft failure and improve outcomes in high-risk patients. There are several surgical techniques described, with quadrupled hamstring grafts being the most commonly used for this type of reconstruction. 5

Many techniques have been described for combined ACL and ALL reconstruction. 6 7 8 9 10 11 12 13 Many of them use one single strand of the gracilis tendon (GT) for ALL reconstruction and the remainder for the ACL. 14 However, using a single strand of the hamstring for ALL reconstruction leaves only a “triple” graft for ACL reconstruction, which could make it weaker since it would be thinner. Studies on isolated intra-articular ACL reconstruction show that hamstring grafts smaller than 8 mm may present a higher risk of failure, but this is not as well-established when combined with extra-articular reconstruction. Helito et al. 15 showed that grafts of 7 mm or less, when combined with ALL reconstruction, can have similar results to those of isolated intra-articular grafts of 8 mm or more. In other words, theoretically, the ideal scenario would be to create a model/technique that enables ALL reconstruction while still providing a graft thick enough for ACL reconstruction.

**Fig. 1:**
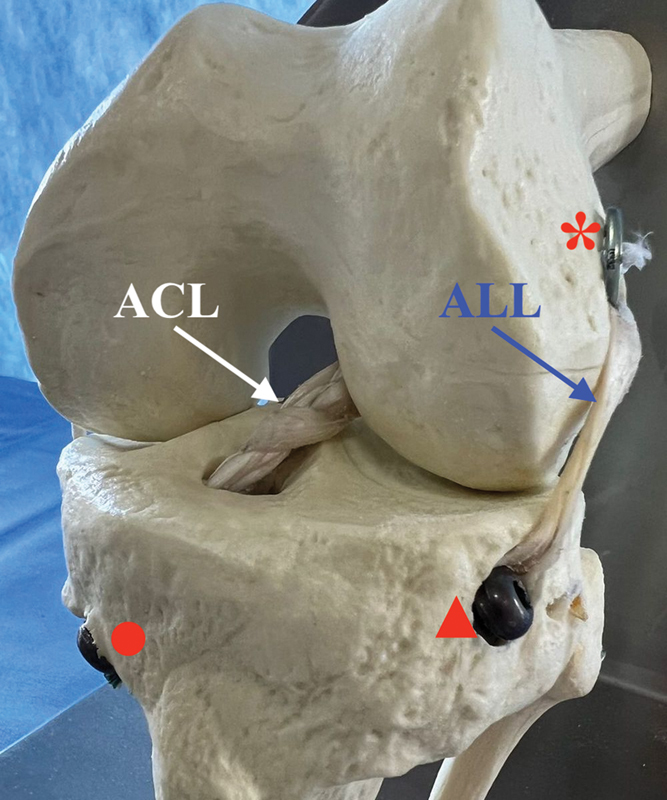
Model demonstrating the technique for anterior cruciate ligament (ACL) and anterolateral ligament (ALL) reconstruction using a triple-braided hamstring graft for the ACL and a single strand for ALL reconstruction. *Endobutton (Smith & Nephew Ltd) securing the triple-braided graft in the femoral tunnel; ▲ interference screw securing the single strand for ALL reconstruction at its tibial insertion, between Gerdy tubercle and the head of the fibula; • interference screw securing the triple-braided graft in the tibial tunnel.

Therefore, the objective of the present study is to describe and biomechanically test a configuration in an animal model that simulates the triple-braided hamstring graft for combined ACL and ALL reconstruction with a single femoral tunnel and a single strand for ALL reconstruction ( Figs. 1 2 ). Our hypothesis is that a triple braid provides a graft thick enough for ACL reconstruction and leaves a single strand of the GT “free” for ALL reconstruction.

**Fig. 2:**
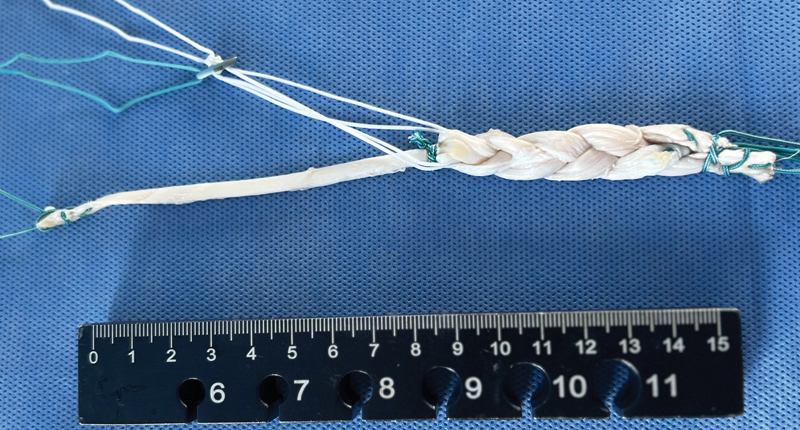
Triple-braided hamstring graft for the ACL with a single strand for ALL reconstruction.

## Materials and Methods

### Test Samples

- To prepare the test samples, a simulated surgery was performed, in which a hole was opened in the polyurethane block, representing the “bone tunnel,” to enable graft insertion and fixation with an interference screw, as would occur in practice.
- According to Brazilian standard ( *norma brasileira* , NBR, in Portuguese) 15678:2020 of Associação Brasileira de Normas Técnicas (Brazilian Association of Technical Standards, ABNT, in Portuguese), which regulates the standard material for the mechanical testing of implants and orthopedic instruments, rigid unicellular polyurethane foam with the following characteristics was applied:
- Dimensions: 100 mm × 100 mm × 30 mm;
- Color: brown;
- Density: 40 pounds per cubic foot (PCF; 0.96 g/cm ^3^ );
- Hole/tunnel: length of 30 mm on the central axis of the 100 mm × 100 mm surface, over the entire height of the block, and a diameter equal to that of the graft.

### Graft

Similarly to what has been described in the biomechanical study by Moré et al., 16 we used recently-frozen Landrace pig legs in the experiments. The tendons were collected from a slaughterhouse. A total of 8 legs were stored at −20°C and thawed 12 hours before the test. Each tibia was dissected and the deep flexor tendon, measuring ∼ 8 mm in width and 9 cm in length, was extracted to be used as graft.

### Sample Preparation

**Fig. 3:**
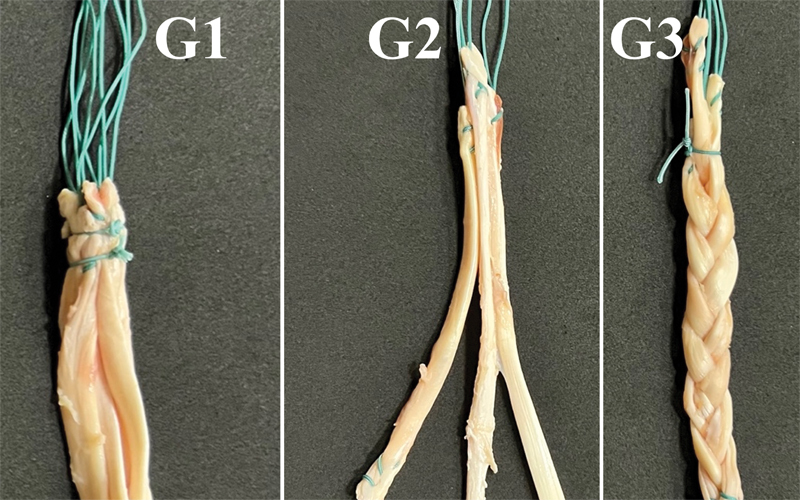
The samples were divided into three groups: group 1–quadruple control group; group 2–simple triple; and group 3–triple-braided.

**Fig. 4:**
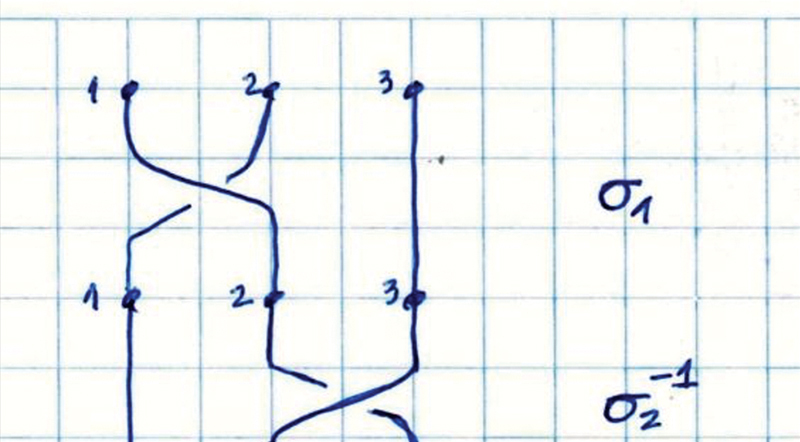
Braiding in a “pure” manner.

The samples were divided into three groups ( Fig. 3 ):

- **Group 1–control Group:** the graft was joined in a quadruple manner and fixed at its ends to the polyurethane blocks with interference screws (made of ASTM F136 titanium alloy, Traumédica Instrumentais e Implantes, Campinas, SP, Brazil); each screw had a length of 30 mm and a diameter equal to that of the graft.
- **Group 2–simple triple:** the graft was joined in a triple parallel manner and fixed at its ends to the polyurethane blocks with interference screws (made of ASTM F136 titanium alloy, Traumédica Instrumentais e Implantes); each screw had a length of 30 mm and a diameter equal to that of the graft diameter.
- **Group 3–triple-braided:** the graft was joined in a triple manner and braided in a “pure” form: (σ1σ2 − 1)3n, with n being a positive integer, meaning the sequence of concatenations σ1σ2 − 1 σ1σ2 − 1 σ1σ2 − 1 repeated an integer number of times ( Fig. 4 ). The basic sequence (σ1σ2 − 1)3n can be correlated with permutations of points (p1, p2, p3) in the following order: (p1, p2, p3), (p2, p1, p3), (p2, p3, p1), (p3, p2, p1), (p3, p1, p2), (p1, p3, p2), and (p1, p2, p3). 17 The braided graft was fixed at its ends to the polyurethane blocks with interference screws (made of ASTM F136 titanium alloy, Traumédica Instrumentais e Implantes); screw had a length of 30 mm and a diameter equal to that of the graft.

The average graft length was of 9 cm, 3 cm inside each block and 3 cm “free” between the blocks. The fixation procedures were performed by a trained orthopedic surgeon. All polyurethane blocks had a tunnel with a diameter equal to that of the graft, which was drilled by the surgeon. The screw was implanted with the aid of a Kirschner wire to avoid divergence and false trajectory. At the end, the test specimens displayed the following configuration: screw – block – graft – block – screw.

### Performance of the Tests

The tests were performed on an EMIC DL 10000 (Instron Brasil Equipamentos Científicos Ltda., São José dos Pinhais, PR, Brazil) electromechanical universal testing machine, using its axial traction to determine the efficiency of graft fixation with interference screws, and a computer to register the data obtained.

In the tests, the experimental length of the sample was correlated by deformation (mm) in relation to time (seconds), stipulated as 10mm− ^2^ /s, with traction applied until graft rupture or slippage of the screw/graft assembly. 18 Nine tests were conducted for each group.

### Methodology and Data Analysis

The categorical and numerical variables were tabulated and analyzed using the R (R Foundation for Statistical Computing, Vienna, Austria) software for Mac OS, which provided measures of central tendency, percentile values, and dispersion.

Data normality was assessed using the Shapiro-Wilk test. The homogeneity of variables among the groups was verified using the Levene test. A comparison of group means, to either reject or accept a null hypothesis, was performed using the *t* -test. The presence of outliers was examined through the development of boxplots. Homoscedasticity was tested through the development of a linear regression model between variables.

Analyses with a 95% confidence interval (95%CI) and a *p* -value lower than 0.05 were considered statistically significant.

## Results

The data obtained from the tests included time (s), deformation (mm), and force (N) to which the samples were subjected. With these data, graphs were drawn of the force (N)/deformation (mm) ratio suffered by the samples fixed with the titanium screws.

In group 1 (quadruple control), the samples achieved a mean peak force of 816.28 ± 78.78 N. As the graft deformation progressed, the force decreased until the graft ruptured, with a mean of approximately 41.30 ± 10.01 mm of deformation relative to the initial length.

In group 2 (simple triple), the samples achieved a mean peak force of 506.95 ± 151.30 N. As the graft deformation advanced, the force decreased until the graft ruptured, with a mean of approximately 36.28 ± 3.25 mm of deformation relative to the initial length.

In group 3 (triple-braided), the samples achieved a mean peak force of 723.16 ± 316.15 N. As the graft deformation advanced, the force decreased until the graft ruptured, with a mean of approximately 52.38 ± 17.35 mm of deformation relative to the initial length.

When comparing the diameter and length of groups 2 and 3, creating a braid in a triple graft increased its diameter by ∼ 9% to 14%. However, this led to a shortening of the graft by ∼ 4% to 8% of its length, with an average peak force increase of ∼ 200 N ( *p* < 0.05), representing an approximate 40% increase in its peak force.

**Fig. 5:**
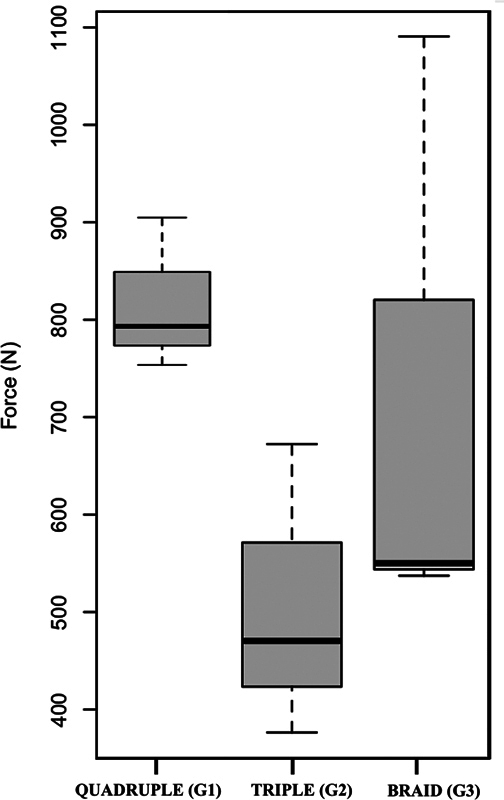
*t* -test regarding peak forces (N) in group 1–(quadruple control), group 2 (simple triple), and group 3 (triple-braided).

Regarding the peak forces in group 1 (quadruple control) and group 2 (simple triple), the *t* -test showed a statistically significant difference between them ( *t* = 3.1452; *p* -value = 0.03467) ( Fig. 5 ). This assessment yielded *p* < 0.05, rejecting the null hypothesis (H0) of no difference between the two groups. In other words, in the study, quadruple and simple triple grafts exhibited different peak forces.

Concerning the peak forces in group 1 (quadruple control) and group 3 (triple-braided), the *t* -test showed no statistically significant difference between them ( *t* = 0.49722; *p* -value = 0.6451) ( Fig. 5 ). This assessment yielded *p* > 0.05, confirming the null hypothesis (H0) of no difference between the two groups. In other words, in the study, quadruple and triple-braided grafts exhibited similar peak forces.

The titanium screw provided secure fixation of the graft in the polyurethane block, without any apparent slippage or deformation. In all samples, the test culminated in graft rupture.

## Discussion

The main finding of the present study demonstrates that the triple-braided graft can be a biomechanically viable alternative when compared with the quadruple graft. The present study opens up new possibilities for multiligament reconstructions, especially for combined reconstructions of the ACL and ALL.

In many medical centers, especially those without access to tissue banks, a significant challenge in ligament reconstructions is graft availability. With a braided configuration, a triple graft can present strength similar to that of a quadruple graft and still leave one single strand available for another reconstruction, such as that of the ALL, for example. From a technical standpoint, braiding is not difficult and only requires a short learning curve.

When three parallel threads are braided, they create a structure that is more resistant and capable of withstanding a variety of forces and conditions, making it a stronger option than individual threads. Braiding improves essential characteristics such as load distribution, torsional resistance, increased contact area, impact absorption, and flexibility. This is frequently used in cables and ropes, and in many other instances in which strength is crucial. 17

When grafts are braided, the load or tension exerted on them is distributed more evenly along the structure. This means that each graft bears a smaller portion of the total load, reducing the risk of rupture. Braiding creates a structure that is more resistant to twisting and bending. If a single graft is bent or twisted, it can break more easily. However, when grafts are braided, they support each other, making the structure more resistant to these forces. Braided grafts have more points of contact with each other than simple parallel threads. This increases the surface area of contact between them, helping distribute stress more effectively and reducing the likelihood of rupture. Braiding also enables the structure to better absorb impacts. When a force is applied to it, the grafts can move within the braid, dissipating the impact energy across the structure rather than concentrating it at a single point. 19

Braiding can provide a certain degree of flexibility to the structure, enabling it to adapt to different conditions and movements without breaking. This is especially useful in applications involving movement or vibrations. However, this is a point that should be noted. In the present study, regarding the peak forces in group 1 (quadruple control) and group 3 (triple-braided), there was no statistically significant difference between them. However, the distribution of peak forces in the samples in group 3 was not as uniform, with some cases exceeding those in group 1 and some approaching those in group 2. Since the braids in group 3 were made manually, we did not standardize their tightness. The tighter the braid, the thicker the graft, providing greater strength but at the cost of greater shortening of its length. 17 19

Graft insufficiency represents one of the main factors determining adverse outcomes in ACL reconstruction. 20 However, there is no solid evidence demonstrating the superiority of autologous grafts compared with other types of grafts. Each graft variety has specific advantages and considerations to consider. Supporters of hamstring tendon grafts have reported a lower incidence of complications in the donor area but increased weakness in hip extension and maximal knee flexion, as well as variable results related to graft size and length, such as a graft diameter shorter than 8 mm, which increases the risk of failure. 21 In many cases, the only available grafts are hamstring tendons, and depending on the patient's body type, the ideal thickness of 8 mm may not be achieved. 20 21

There are numerous studies on graft preparation techniques for ACL reconstruction. Conte et al. 21 suggest that grafts smaller than 8 mm in diameter have high failure rates, and according to Figueroa et al., 22 an increase in graft diameter of just 0.5 mm can lead to statistically significant improvements in graft success and longevity.

Authors like Park et al. 23 and Samitier and Vinagre 24 have reported a technique involving the braiding of four strands of hamstring autograft. According to these authors, braiding a 4-strand hamstring autograft can increase graft diameter by around 1 mm to 1.5 mm, but may result in a shortening of ∼ 5 mm to 10 mm. Therefore, this technique is not recommended for very short grafts.

Other theoretical advantages of the hamstring autograft braiding technique include obtaining a uniform graft strip that appears to mimic the native shape of the ACL and replicate its mechanical behavior, 25 as well as compensate for the intrinsic viscoelasticity associated with soft tissue grafts, minimizing postreconstruction stretching that can lead to laxity and reruptures. 24

Regarding techniques for ACL and ALL reconstruction, Helito et al. 14 used a quadruple graft, combining three strands of the semitendinosus tendon (ST) and one of the gracilis tendon for the ACL and a single strand of the GT for the ALL; tibial fixation was performed with anchors. Sonnery-Cottet et al. 26 used a triple ST graft for the ACL and a double GT graft for the ALL, with two tibial tunnels for ALL reconstruction.

Ferreira et al. 11 employed a graft preparation method that creates a suspension effect similar to that of the Endobutton (Smith & Nephew Ltd., London, United Kingdom), adding a suture to reinforce this union, and including an interference screw. This enables the end of the ST graft to remain close to the femoral joint point, and it does not need to occupy the entire tunnel, which will be completed by the GT graft, facilitating the procedure for short grafts. This technique is similar to what has been described in the present study, with the exception of the triple braid.

Therefore, it is essential to master various graft preparation techniques to obtain an individualized graft with the appropriate diameter and length that match the patient's anatomy, height, and physical demands. The triple-braided hamstring autograft technique is a reliable graft configuration, relatively easy to prepare, and reproducible, providing a stronger and more uniform hamstring graft.

## Limitations

The major limitation of the present study was the choice of graft for testing. Due to the ease of obtaining it, we used animal tissue as the graft; however, pig grafts do not have the same strength as young human grafts. Thus, we could not test the full capacity of the screw-polyurethane-graft complex. Nevertheless, the methodology followed in the present study is a useful model for future research. Another limitation was that the braids were made manually, meaning there was no standardization in terms of their tightness. Despite these limitations, our results are consistent with those obtained in other similar studies. Overall, the results indicate that the proposed configuration results in acceptable biomechanical performance. However, more research is needed to determine the clinical relevance of these findings.

## Conclusion

The triple-braided hamstring graft configuration for combined ACL and ALL reconstruction with a single femoral tunnel and a single strand for ALL reconstruction may become a biomechanically viable solution, with potential clinical application.
