# Supplementary material for: Analysis of the Biomechanical Behavior of an Animal Model of Triple Hamstring Graft Configuration for Combined ACL and ALL Reconstruction with a Single Femoral Tunnel and a Single Strand for ALL Reconstruction
Source: Rev Bras Ortop (Sao Paulo). 2024 May 19;59(4):e549–55. doi: 10.1055/s-0044-1785664 (PMC11374408; doi:10.1055/s-0044-1785664)
Supplement: Related texts

# Research article: Análise do comportamento biomecânico de um modelo animal de configuração de enxerto de trança tripla de isquiotibiais para reconstrução combinada de LCA e LAL para túnel único femoral

**Keywords:** articulação do joelho, ligamento cruzado anterior, ligamentos articular, reconstrução do ligamento cruzado anterior

## Abstract

**Objetivo** Descrever e testar biomecanicamente uma configuração, num modelo animal que simula o enxerto triplo de isquiotibiais para a reconstrução combinada do ligamento cruzado anterior (LCA) e do ligamento anterolateral (LAL), com apenas um túnel femoral e somente uma “perna” para a reconstrução do LAL.

**Métodos** Tendões flexores profundos de suínos foram utilizados como enxerto e fixados com parafusos de interferência de titânio num bloco de poliuretano. As amostras foram divididas em 3 grupos: grupo 1 (controle) – com enxerto quádruplo; grupo 2 – com enxerto triplo simples; e grupo 3–com enxerto triplo trançado. Os testes foram realizados com uma máquina universal de ensaios eletromecânica EMIC DL 10000 (Instron Brasil Equipamentos Científicos Ltda., São José dos Pinhais, PR, Brasil).

**Resultados** As amostras dos grupos 1, 2 e 3 alcançaram forças de pico médias de 816,28 ± 78,78 N, 506,95 ± 151,30 N e 723,16 ± 316,15 N, respetivamente. No Grupo 3, o trançado aumentou o diâmetro do enxerto entre 9% e 14%, e causou um encurtamento de 4% a 8% em comparação ao grupo 1, com um aumento médio da força de pico de ∼ 200 N ( *p* < 0,05). Não houve diferença estatisticamente significativa entre os grupos 1 e 3 com relação às forças de pico, o que indica que os enxertos quádruplos e triplos trançados apresentaram resultados de força semelhantes.

**Conclusão** A configuração do enxerto de isquiotibiais com trança tripla para a reconstrução combinada do LCA e do LAL com um só túnel femoral e uma só “perna” para a reconstrução do LAL pode se tornar uma solução biomecanicamente viável, com potencial aplicação clínica.

## Introdução

A reconstrução combinada do ligamento cruzado anterior (LCA) e do ligamento anterolateral (LAL) do joelho tem mostrado excelentes resultados em grupos específicos de pacientes. 1 2 3 4 Essa reconstrução poderia reduzir a falha do enxerto e melhorar os resultados em pacientes de alto risco. Existem várias técnicas cirúrgicas descritas, sendo os enxertos de isquiotibiais quádruplo os mais utilizados para este tipo de reconstrução. 5

Muitas técnicas têm sido descritas para a reconstrução combinada do LCA e do LAL, 6 7 8 9 10 11 12 13 e muitas delas utilizam apenas uma “perna” do tendão do músculo grácil (TG) para a reconstrução da LAL, e o restante, para o LCA. 14 No entanto, a utilização de somente uma “perna” do tendão do grácil para a reconstrução do LAL deixa apenas um enxerto “triplo” para a reconstrução do LCA, o que poderia torná-lo mais fraco, uma vez que seria mais fino. Estudos sobre reconstrução intra-articular isolada do LCA mostram que enxertos de isquiotibiais menores do que 8 mm podem ter um risco maior de falha, mas isso não é tão bem estabelecido quando combinado com reconstrução extra-articular. Helito et al. 15 mostraram que enxertos de 7 mm ou menos, quando combinados com reconstrução do LCA, podem ter resultados similares aos dos enxertos intra-articulares isolados de 8 mm ou mais. Em outras palavras, teoricamente, o cenário ideal seria criar um modelo/técnica que permitisse a reconstrução LAL e, ao mesmo tempo, proporcionasse um enxerto suficientemente espesso para a reconstrução do LCA.

Sendo assim, o objetivo deste estudo é descrever e testar biomecanicamente uma configuração, em um modelo animal que simula o enxerto de trança tripla de tendões dos isquiotibiais para a reconstrução combinada do LCA e da LAL, com apenas um túnel femoral e somente uma “perna” para a reconstrução da LAL ( Figs. 1 2 ). A nossa hipótese é a de que uma trança tripla proporciona um enxerto suficientemente espesso para a reconstrução do LCA, e deixa uma “perna” do TG “livre” para a reconstrução da LAL.

**Fig. 1:** Modelo demonstrativo da técnica de reconstrução do ligamento cruzado anterior (LCA) e do ligamento anterolateral (LAL) utilizando um enxerto de isquiotibiais triplamente trançado para o LCA e apenas uma “perna” para a reconstrução do LAL. * Endobutton (Smith & Nephew Ltd.) que fixa o enxerto triplamente trançado no túnel femoral; ▲ Parafuso de interferência que fixa a “perna” única do tendão do grácil para a reconstrução de LAL na sua inserção tibial entre o tubérculo de Gerdy e a cabeça da fíbula; • Parafuso de interferência que fixa o enxerto triplamente trançado no túnel tibial.

**Fig. 2:** Enxerto de isquiotibiais com trança tripla para o LCA somente com um fio para a reconstrução do LAL.

## Materiais e métodos

## Amostras de teste

Para preparar as amostras de teste, foi realizada uma simulação de cirurgia em que foi aberto um orifício no bloco de poliuretano, que representava o “túnel ósseo”, para permitir a inserção do enxerto e fixá-lo com um parafuso de interferência, como ocorreria na prática.

De acordo com a norma brasileira (NBR) 15678:2020 da Associação Brasileira de Normas Técnicas (ABNT), que regulamenta o material-padrão para o ensaio mecânico de implantes e instrumentais ortopédicos, foi aplicada uma espuma rígida de poliuretano unicelular com as seguintes características:

- Dimensões: 100 mm × 100 mm × 30 mm;
- Cor: marrom;
- Densidade: 40 libras por pé cúbico (lb/ft ^3^ ; – 0,96 g/cm ^3^ );
- Orifício/túnel: comprimento de 30 mm no eixo central da superfície de 100 mm × 100 mm, em toda a altura do bloco, e um diâmetro igual ao do enxerto.

## Enxerto

Semelhante ao que foi descrito no estudo biomecânico de Moré et al., 16 utilizamos pernas de porco Landrace recentemente congeladas nas experiências. Os tendões foram colhidos em um matadouro. Ao todo, 8 patas foram armazenadas a −20°C e descongeladas 12 horas antes do ensaio. Cada tíbia foi dissecada e o tendão flexor profundo, com ∼ 8 mm de largura e 9 cm de comprimento, foi extraído para ser utilizado como enxerto.

## Preparação da amostra

As amostras foram divididas em três grupos ( Fig. 3 ):

**– Grupo 1–grupo de controle:** o enxerto foi unido de forma quádrupla e fixado nas suas extremidades aos blocos de poliuretano com parafusos de interferência (de liga de titânio ASTM F136, Traumédica Instrumentais e Implantes, Campinas, SP, Brasil); cada parafuso tinha comprimento de 30 mm e diâmetro igual ao do enxerto.
**– Grupo 2–triplo simples:** o enxerto foi unido de forma triplamente paralela e fixado em suas extremidades aos blocos de poliuretano com parafusos de interferência (de liga de titânio ASTM F136, Traumédica Instrumentais e Implantes); cada parafuso tinha comprimento de 30 mm e diâmetro igual ao do enxerto.
**– Grupo 3–trançado triplo:** o enxerto foi unido de forma tripla e trançado de forma “pura”: (σ1σ2-1)3n, sendo n um número inteiro positivo, que significa a sequência de concatenações σ1σ2-1 σ1σ2-1 σ1σ2-1 repetida um número inteiro de vezes ( Fig. 4 ). A sequência básica (σ1σ2-1)3n pode ser correlacionada com permutações de pontos (p1, p2, p3) na seguinte ordem: (p1, p2, p3), (p2, p1, p3), (p2, p3, p1), (p3, p2, p1), (p3, p2, p1), (p3, p1, p2), (p1, p3, p2), e (p1, p2, p3). 17 O enxerto trançado foi fixado em suas extremidades aos blocos de poliuretano com parafusos de interferência (de liga de titânio ASTM F136, Traumédica Instrumentais e Implantes); cada parafuso tinha comprimento de 30 mm e diâmetro igual ao do enxerto.

**Fig. 3:** As amostras foram divididas em três grupos: grupo 1–grupo de controle quádruplo; grupo 2–triplo simples; e grupo 3–trançado triplo.

**Fig. 4:** Trançar de uma forma “pura”.

O comprimento médio do enxerto foi de 9 cm, com 3 cm dentro de cada bloco e 3 cm “livres” entre os blocos. Os procedimentos de fixação foram realizados por um cirurgião ortopédico qualificado. Todos os blocos de poliuretano tinham um túnel com diâmetro igual ao do enxerto, que foi perfurado pelo cirurgião. O parafuso foi implantado com o auxílio de um fio de Kirschner para evitar divergência e falsa trajetória. No final, os corpos de prova apresentavam a seguinte configuração: parafuso – bloco – enxerto – bloco – parafuso.

## Realização dos testes

Os testes foram realizados numa máquina eletromecânica de ensaios universais EMIC DL 10000 (Instron Brasil Equipamentos Científicos Ltda., São José dos Pinhais, PR, Brasil), utilizando a sua tração axial para determinar a eficácia da fixação do enxerto com parafusos de interferência e um computador para registar os dados obtidos.

Nos testes, o comprimento experimental da amostra foi correlacionado pela deformação (mm) em relação ao tempo (segundos), estipulado em 10 mm− ^2^ /s, com tração aplicada até a ruptura do enxerto ou deslizamento do conjunto parafuso/enxerto. 18 Foram realizados nove testes para cada grupo.

## Metodologia e análise de dados

As variáveis categóricas e numéricas foram tabuladas e analisadas no programa R (R Foundation for Statistical Computing, Viena, Áustria) para Mac OS, que forneceu medidas de tendência central, valores de percentil e dispersão.

A normalidade dos dados foi avaliada por meio do teste de Shapiro-Wilk. A homogeneidade das variáveis entre os grupos foi verificada por meio do teste de Levene. A comparação das médias dos grupos para rejeitar ou aceitar uma hipótese nula foi efetuada utilizando o teste *t* . A presença de *outliers* foi verificada através da construção de *boxplots* . A homocedasticidade foi testada por meio do desenvolvimento de um modelo de regressão linear entre as variantes.

As análises com intervalo de confiança de 95% (IC95%) e valor de *p* inferior a 0,05 foram consideradas estatisticamente significativas.

## Resultados

Os dados obtidos nos testes incluíram o tempo (s), a deformação (mm) e a força (N) a que as amostras foram sujeitas. A partir desses dados, foram feitos gráficos da relação força (N)/deformação (mm) sofrida pelas amostras fixadas com os parafusos de titânio.

No grupo 1 (controle quádruplo), as amostras atingiram uma média de pico de força de 816,28 ± 78,78 N. A partir da progressão da deformação do enxerto, a força diminuiu até atingir a rutura do enxerto, com média de aproximadamente 41,30 ± 10,01 mm de deformação relativamente ao comprimento inicial.

No grupo 2 (triplo simples), as amostras atingiram uma média de pico de força de 506,95 ± 151,30 N. Na medida em que a deformação do enxerto avançava, a força foi diminuindo até atingir a ruptura do enxerto, com média de aproximadamente 36,28 ± 3,25 mm de deformação relativamente ao comprimento inicial.

No grupo 3 (trançado triplo), as amostras atingiram uma média de pico de força de 723,16 ± 316,15 N. Com o avanço da deformação do enxerto, a força diminuiu até atingir a ruptura do enxerto, com média de aproximadamente 52,38 ± 17,35 mm de deformação relativamente ao comprimento inicial.

Ao comparar o diâmetro e o comprimento dos grupos 2 e 3, a criação de uma trança num enxerto triplo aumentou o seu diâmetro em ∼9% a 14%. No entanto, houve encurtamento do enxerto em ∼4% a 8% do seu comprimento, com um aumento médio da força de pico de ∼200 N ( *p* < 0,05), o que representa um aumento aproximado de 40%.

Em relação ao pico de força, o teste *t* t mostrou diferença estatisticamente significativa entre os grupos 1 e 2 ( *t* = 3,1452; *p* = 0,03467) ( Fig. 5 ). Nessa avaliação, obtivemos *p* < 0,05, o que significa rejeição da hipótese nula (H0) de que não há diferença entre os dois grupos. Em outras palavras, os enxertos quádruplos e triplos simples apresentaram picos de força diferentes neste estudo.

**Fig. 5:** Teste *t* relativo às forças de pico (N) do grupo 1 (controle quádruplo), grupo 2 (triplo simples) e grupo 3 (trançado triplo).

Ainda com relação ao pico de força, o teste *t* não apresentou diferença estatisticamente significativa entre os grupo9s 1 e 3 ( *t* = 0,49722; *p* = 0,6451) ( Fig. 5 ). Nessa avaliação, obtivemos *p* > 0,05, o que confirma a hipótese nula (H0) de que não há diferença entre os dois grupos. Neste estudo, portanto, os enxertos quádruplos e triplos trançados apresentaram picos de força semelhantes.

O parafuso de titânio proporcionou uma fixação segura do enxerto no bloco de poliuretano, sem qualquer deslizamento ou deformação aparente. Em todas as amostras, o teste culminou com a ruptura do enxerto.

## Discussão

O achado principal do presente estudo demonstra que o enxerto triplo trançado pode ser uma alternativa biomecanicamente viável quando comparado ao enxerto quádruplo. Este estudo abre novas possibilidades para as reconstruções multiligamentares, em especial no que se refere às reconstruções combinadas do LCA e do LAL.

Em muitos centros, especialmente aqueles sem acesso a bancos de tecidos, um desafio significativo nas reconstruções ligamentares é a disponibilidade do enxerto. Com uma configuração trançada, um enxerto triplo pode ter uma resistência semelhante à de um enxerto quádruplo, e ainda ter centros médicos disponíveis para outra reconstrução, como a do LAL, por exemplo. Do ponto de vista técnico, o trançado não é difícil, e requer apenas uma curta curva de aprendizagem.

Quando três fios paralelos são trançados, eles criam uma estrutura que é mais resistente e capaz de suportar uma variedade de forças e condições, sendo uma opção mais resistente do que os fios individuais. O trançado melhora características essenciais, como a distribuição da carga, a resistência à torção, o aumento da área de contato, a absorção do impacto e a flexibilidade. É frequentemente utilizado em cabos, cordas, e muitas outras situações em que a resistência é crucial. 17

Quando os enxertos são trançados, a carga ou tensão exercida sobre eles é distribuída de forma mais uniforme ao longo da estrutura. Isto significa que cada enxerto suporta uma porção menor da carga total, o que reduz o risco de ruptura. O trançado cria uma estrutura mais resistente à torção e à flexão. Se somente um enxerto for dobrado ou torcido, ele pode quebrar mais facilmente. No entanto, quando os enxertos são trançados, eles se apoiam uns nos outros, o que torna a estrutura mais resistente a essas forças. Os enxertos trançados têm mais pontos de contato uns com os outros do que os fios paralelos simples, o que aumenta a área de superfície de contato entre eles, ajuda a distribuir a tensão de forma mais eficaz e reduz a probabilidade de ruptura. O trançado também permite que a estrutura absorva melhor os impactos. Quando aplicada uma força, os enxertos podem se mover dentro do trançado e dissipar a energia do impacto por toda a estrutura, em vez de concentrá-la em um só ponto. 19

O trançado pode conferir um certo grau de flexibilidade à estrutura, o que possibilita a adaptação a diferentes condições e movimentos sem que ela se quebre, o que é especialmente útil em aplicações com movimento ou vibrações. É necessário, no entanto, observar este ponto com atenção. No presente estudo, não obtivemos uma diferença estatisticamente significativa em relação aos picos de força do grupo 1 (quádruplo) e do grupo 3 (triplo trançado). Por outro lado, a distribuição das forças de pico nas amostras do grupo 3 não foi tão uniforme, com alguns casos excedendo a do grupo 1, e outros, aproximando-se do Grupo 2. Como as tranças do grupo 3 foram feitas manualmente, não padronizamos o seu grau de aperto. Quanto mais firme for a trança, mais espesso será o enxerto, o que proporciona maior resistência e provoca um maior encurtamento do seu comprimento. 17 19

A insuficiência do enxerto representa um dos principais fatores que determinam resultados adversos na reconstrução do LCA. 20 No entanto, não há evidências sólidas que demonstrem a superioridade dos enxertos autólogos em relação a outros tipos de enxerto. Cada variante de enxerto tem vantagens específicas e considerações específicas a serem consideradas. Os adeptos dos enxertos de tendões isquiotibiais relatam uma incidência menor de complicações na área doadora, mas uma maior fraqueza na extensão do quadril e na flexão máxima do joelho, bem como resultados variados em relação ao tamanho e ao comprimento do enxerto; por exemplo, o risco de insucesso aumenta com um enxerto de diâmetro inferior a 8 mm. 21 Em muitos casos, os únicos enxertos disponíveis são os de tendões dos isquiotibiais, e, dependendo do tipo de corpo do paciente, a espessura ideal de 8 mm pode não ser alcançada. 20 21

Existem inúmeros estudos sobre técnicas de preparação de enxertos para a reconstrução do LCA. Conte et al. 21 sugerem que os enxertos com menos de 8 mm de diâmetro têm elevadas taxas de insucesso e, de acordo com Figueroa et al., 22 um aumento do diâmetro do enxerto de apenas 0,5 mm pode levar a melhoras estatisticamente significativas no sucesso e na longevidade do enxerto.

Autores como Park et al. 23 e Samitier e Vinagre 24 relataram uma técnica que envolve o trançado de quatro fios de autoenxerto de isquiotibiais. Segundo os autores, trançar um autoenxerto de isquiotibiais de 4 fios pode aumentar o diâmetro do enxerto em cerca de 1 mm a 1,5 mm, mas isso pode resultar em um encurtamento de ∼ 5 mm a 10 mm. Portanto, a técnica não é recomendada para enxertos muito curtos.

Outras vantagens teóricas da técnica de trançado do autoenxerto de isquiotibiais incluem a obtenção de uma tira de enxerto uniforme, que parece imitar a forma nativa do LCA e replicar o seu comportamento mecânico, 25 e também compensa a viscoelasticidade intrínseca associada aos enxertos de tecidos moles, o que minimiza o estiramento pós-reconstrução e pode levar à frouxidão e a novas rupturas. 24

Em relação às técnicas de reconstrução do LCA e do LAL, Helito et al. 14 utilizaram um enxerto quádruplo, triplo do tendão do músculo semitendíneo (TS) e uma “perna” do tendão do músculo grácil para o LCA, e apenas uma única “perna” do TG para o LAL; a fixação tibial foi realizada com âncoras. Sonnery-Cottet et al. 26 utilizaram um enxerto triplo de TS para o LCA e um enxerto duplo de TG para o LAL, com dois túneis tibiais para a reconstrução do LAL.

Ferreira et al. 11 utilizaram um método de preparação do enxerto que cria um efeito de suspensão semelhante ao do Endobutton (Smith & Nephew Ltd., Londres, Reino Unido), ao adicionar uma sutura para reforçar a união e incluir um parafuso de interferência. Isso permite que a extremidade do enxerto do TS permaneça próxima ao ponto articular femoral, não necessitando ocupar todo o túnel, que será preenchido pelo enxerto do TG, o que facilita o procedimento para enxertos curtos. Essa técnica é semelhante à descrita neste estudo, com exceção da trança tripla.

Por isso, é essencial dominar várias técnicas de preparação do enxerto para obter um enxerto individualizado com o diâmetro e comprimento adequados à anatomia, altura e exigências físicas do paciente. A técnica de autoenxerto de isquiotibiais com trança tripla é uma configuração de enxerto confiável, relativamente fácil de preparar e reproduzível, que resulta em um enxerto de isquiotibiais mais forte e uniforme.

## Limitações

A maior limitação deste estudo foi a escolha do enxerto para o teste. Devido à facilidade de obtenção, utilizamos um tecido animal como enxerto. No entanto, os enxertos de porco não têm a mesma resistência do que os enxertos humanos jovens. Dessa forma, não foi possível testar a capacidade total do complexo parafuso-poliuretano-enxerto. A metodologia seguida na realização deste estudo é um modelo útil para pesquisas futuras. Outra limitação foi o fato de as tranças terem sido feitas manualmente, o que significa que não houve uma padronização do grau de aperto das tranças. Apesar dessas limitações, os nossos resultados são consistentes com os obtidos em outros estudos semelhantes. Em geral, os resultados indicam que a configuração proposta proporciona um desempenho biomecânico aceitável. No entanto, é preciso realizar outras investigações para determinar a relevância clínica dos resultados.

## Conclusão

A configuração do enxerto de isquiotibiais com trança tripla para a reconstrução combinada do LCA e do LAL com apenas um túnel femoral e somente uma “perna” para a reconstrução do LAL pode se tornar uma solução mecanicamente viável, com potencial aplicação clínica.
